# The role of thrombin in early-onset seizures

**DOI:** 10.3389/fncel.2023.1101006

**Published:** 2023-03-09

**Authors:** Alina Savotchenko, Mariia Klymenko, Mariia Shypshyna, Dmytro Isaev

**Affiliations:** ^1^Department of Cellular Membranology, Bogomoletz Institute of Physiology, Kyiv, Ukraine; ^2^Laboratory of Synaptic Transmission, Bogomoletz Institute of Physiology, Kyiv, Ukraine

**Keywords:** thrombin, blood-brain barrier, hippocampus, status epilepticus, temporal-lobe epilepsy, lithium-pilocarpine model

## Abstract

A variety of clinical observations and studies in animal models of temporal lobe epilepsy (TLE) reveal dysfunction of blood-brain barrier (BBB) during seizures. It is accompanied by shifts in ionic composition, imbalance in transmitters and metabolic products, extravasation of blood plasma proteins in the interstitial fluid, causing further abnormal neuronal activity. A significant amount of blood components capable of causing seizures get through the BBB due to its disruption. And only thrombin has been demonstrated to generate early-onset seizures. Using the whole-cell recordings from the single hippocampal neurons we recently showed the induction of epileptiform firing activity immediately after the addition of thrombin to the blood plasma ionic media. In the present work, we mimic some effects of BBB disruption *in vitro* to examine the effect of modified blood plasma artificial cerebrospinal fluid (ACSF) on the excitability of hippocampal neurons and the role of serum protein thrombin in seizure susceptibility. Comparative analysis of model conditions simulating BBB dysfunction was performed using the lithium-pilocarpine model of TLE, which most clearly reflects the BBB disruption in the acute stage. Our results demonstrate the particular role of thrombin in seizure-onset in conditions of BBB disruption.

## 1. Introduction

Breakdown of the BBB is a most common feature of brain disorders, accompanied by neural and network dysfunction and degeneration (Benveniste et al., 1984; Seiffert, 2004; Tomkins et al., 2008), including epilepsy, stroke, traumatic brain injury, tumors, and neurodegenerative diseases (Brown and Davis, 2002; Davies, 2002; van Vliet et al., 2007; Stolp and Dziegielewska, 2009; Chodobski et al., 2011; Vezzani and Friedman, 2011; On et al., 2013; Wu et al., 2020). Studies on animal models of epilepsy and clinical observations among human patients reveal that the BBB has a direct role in epileptogenesis and brain damage (Mihály and Bozóky, 1984; Oby and Janigro, 2006; Friedman, 2011; Greene et al., 2022). Status epilepticus (SE) is accompanied by endothelial impairment and increased blood vessel permeability, which results in a disbalance of the neuronal environment (Obermeier et al., 2013). In particular, the ionic composition of the intercellular cerebrospinal fluid in the involved tissues is close in concentration to blood plasma (Zauner et al., 1996; Reinert et al., 2000). Such shifts in the interstitial ions can lead to changes in the impulse activity of neurons and affect the efficiency of synaptic transmission and, as a result, contribute to an increase in excitability of hippocampal neural networks (Rasmussen et al., 2020). Extravasation of blood plasma proteins into the extracellular environment of the brain in case of BBB damage also contributes to long-term hypersynchronization of neurons in the affected areas (Seiffert, 2004; van Vliet et al., 2007). Entering the brain tissue as a consequence of traumatic brain injury thrombin is able to induce seizures (Lee et al., 1997). *In vitro* studies have shown the enhancement of thrombin activity in the brain due to pilocarpine treatment (Golderman et al., 2019). Recent findings suggest a significant increase in thrombin level in the brain following SE (Isaev et al., 2015). Moreover, intracerebral injection of thrombin may directly induce seizures (Lee et al., 1997). In this work, we simulate certain conditions of BBB breakdown *in vitro* to study the effect of thrombin in blood plasma ionic media on induction of epileptiform activity in hippocampal slices. Using the classical model of TLE we have found the resemblance in the manifestation of early-life seizures in SE-treated rats compared to seizure-like activity (SLA) due to model conditions, simulating impairment of BBB *in vitro*.

## 2. Materials and methods

### 2.1. Animals and experimental design

Experiments were conducted as per international principles of the European Convention for the protection of vertebrate animals used for experimental and other scientific purposes (European convention, Strasburg, 1986); the Law of Ukraine “On protection of animals from cruelty” and approved by the Animal Care Committee of Bogomoletz Institute of Physiology.

In our study, we use two groups of animals: control and SE-treated male Wistar rats at postnatal day (P) 21. The age was chosen based on brain sensitivity to pilocarpine (Cavalheiro et al., 1987). Hippocampal slices of control rats were subdivided into two groups: first for investigating the effect of modified blood plasma solution alone on induction of epileptiform activity (*n* = 17) and second—for the estimation the influence of the same solution together with 5 U/ml thrombin (*n* = 18) in order to mimic BBB disruption *in vitro*. Slices of SE-treated rats (*n* = 14) were used to compare their seizure-like activity with that of control hippocampi, induced by thrombin in blood plasma saline. For SE initiation rats were exposed to intraperitoneal injection (i.p.) of lithium chloride (127 mg/kg, 1 ml/kg) 20–22 h before administration of pilocarpine (i.p). First rats received one 40 mg/kg dose of pilocarpine with the subsequent injection of an additional 10 mg/kg dose every 30 min until the SE induction. The maximal pilocarpine concentration was 60 mg/kg per animal. We set the start of SE when the rat reached Racine stage V seizures (Racine, 1972) and terminate it at 60 min after onset by diethyl ether. Immediately after the animal fell asleep, we prepared hippocampal slices for further electrophysiological studies.

### 2.2. Hippocampal slice preparation

Upon anesthesia by diethyl ether and rapid decapitation, the brain was removed and placed into ice-cold carbogenated (5% CO_2_ and 95% O_2_) artificial cerebrospinal fluid (ACSF) containing (in mM): 119 NaCl, 2.5 KCl, 2 CaCl_2_, 1.3 MgCl_2_, 26 NaHCO_3_, 1 NaH_2_PO_4_, and 11 glucose, pH 7.35. Isolated hippocampi were cut into 500 μm slices with a Vibroslice NVSL (World Precision Instruments Inc., Sarasota, FL, USA), and maintained in a carbogenated ACSF at room temperature for at least 1.5 h before recordings.

### 2.3. Electrophysiological procedure

Extracellular field potential recordings were made with glass microelectrodes containing ACSF (resistance of 1–3 MΩ) placed in the stratum pyramidale CA1. Brain slices were continuously superfused at a rate of 2–4 ml/min with carbogenated ASCF (30–32°C). Signals were digitized using an analog-to-digital converter (NI PCI-6221, National Instruments, Austin, TX, USA) and stored on a computer with WinWCP software (Strathclyde Electrophysiology Software, University of Strathclyde, Glasgow, UK).

To mimic some effects of BBB dysfunction we used an ASCF adapted to the blood plasma ionic media (in mM): 125 NaCl, 5 KCl, 1 CaCl_2_, 0.8 MgCl_2_, 24 NaHCO_3_, 1.25 NaH_2_PO_4_, and 11 glucose, pH 7.35; Katzman and Pappius, 1973). Field potential recordings of SLA induced in blood plasma ACSF were performed on 17 slices (nine rats). Application of 5 U/ml thrombin to the blood plasma ionic media enhance seizure occurrence (18 slices, eight rats). Electrophysiological studies on hippocampal slices of SE-treated rats (14 slices, nine rats) were performed in incubation ASCF. No more than three slices per each animal were used.

### 2.4. Data analysis

Off-line data analysis was performed using Clampfit (Axon Instruments, CA, USA), Origin 7.5 (OriginLab, Northampton, MA, USA), and GraphPad Prism 5 (GraphPad, MA, USA) software. SLA was defined as brief, high amplitude spikes in the EEG. The Kruskal-Wallis test and* post hoc* (Dunn) were used for statistical comparison across groups. Data are shown as mean ± SEM.

## 3. Results

**Figure 1:**
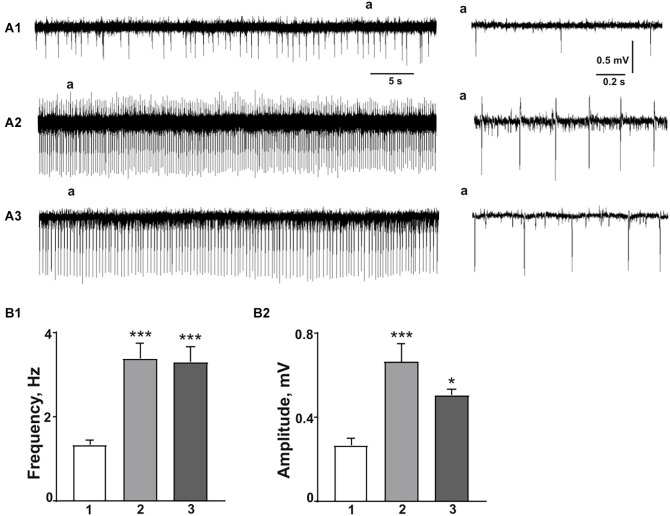
Effect of thrombin in plasma ionic media on the induction of SLA of CA1 subfield compared to Li-pilocarpine seizures. **(A1)** Extracellular field potentials recorded from CA1 pyramidal cell layer in modified to blood plasma ASCF indicate the induction to epileptiform activity. **(A2)** Application of 5U/ml thrombin produces robust SLA. **(A3)** Ictal-like events in SE-treated slices are similar to those, obtained in the presence of thrombin in blood plasma saline. Spontaneous discharges (a) shown in expanded scales in the right panel. Summary plots show the SLA frequency **(B1)** and amplitude **(B2)** during epileptiform discharges in blood plasma media (1), after the application of thrombin (2), and in SE-treated slices (3). Data presented as mean ± SEM. *P < 0.01, ***P < 0.0001.

Field potential recordings were performed from the hippocampal CA1 pyramidal layer in acute slices. We did not observe SLA appearance in any of the tested slices due to ACSF perfusion (data not shown). Bath application of blood plasma ionic media led to consistent SLA in 17 slices (Figure 1A1). This activity persisted as long as examined solution was applied. The frequency of synchronous discharges during SLA was 1.34 ± 0.11 Hz. The amplitude of SLA was at the level 0.27 ± 0.03 mV (Figure 1B). Addition of 10 U/ml thrombin alone did not lead to an increase in neuronal firing in the CA3 hippocampal region of P 6–15 rats (Isaeva et al., 2012). Thrombin has evoked SLA in the presence of 7.2 mM of K^+^ or 100 μM of glutamate in the extracellular solution (Maggio et al., 2008). In our study application of 5 U/ml thrombin together with blood plasma ionic saline induced SLA in hippocampal CA1 pyramidal layer with the frequency of 3.39 ± 0.35 Hz and amplitude 0.66 ± 0.09 mV (*n* = 18, Figure 1A2). These data are in agreement with our previous report when in similar conditions the epileptiform activity in cultured hippocampal neurons was significantly enhanced (Shypshyna et al., 2021). Extracellular recordings from the slices of SE-treated rats demonstrated high-frequency oscillations with the frequency of 3.31 ± 0.35 Hz and amplitude 0.51 ± 0.03 mV (*n* = 14, Figure 1A3). Comparative analysis of field potentials in all experimental groups reveal significant enhancement in frequency (*H*_(2)_ = 26.74, *P* < 0.0001; post-hoc: blood plasma media vs. thrombin—*P* < 0.0001; blood plasma media vs. SE—*P* < 0.0001, Figure 1B1) and amplitude (*H*_(2)_ = 20.30, *P* = 0.0001; *post-hoc*: blood plasma media vs. thrombin—*P* < 0.0001; blood plasma media vs. SE—*P* = 0.0011, Figure 1B2) of SLA due to thrombin application and SE exposure. We observed non-significant decrease in amplitude of SLA in slices obtained from Li-pilocarpine-exposed epileptic rats compared to thrombin-treated slices in blood-plasma ACSF (*p* = 0.99, Figure 1B2).

## 4. Discussion

The main finding of our study is that TLE-treated slices shortly after SE induction produces similar significant increase in frequency and amplitude of SLA as blood plasma media together with thrombin.

Acute SE is induced by systemic application of muscarinic agonist, pilocarpine. *In vitro* application of pilocarpine alone did not cause epileptiform activity, but induced seizures when applied with substances that enhance BBB leakage, such as bradykinin or histamine (Uva et al., 2008). To promote an increase of BBB permeability we first perform the *in vivo* injection of lithium chloride. Subsequent administration of pilocarpine induced SE.

In our recent study, the application of serum-adapted solution increases the average action potentials frequency in neurons with spontaneous firing activity as well as tonic electrical activity in neurons. Increasing neuronal activity by blood plasma ACSF led to the development of epileptiform tonic activity in cultured hippocampal neurons (Shypshyna et al., 2021). Thrombin was shown to facilitate the effects of proconvulsants in the hippocampal slices from adult rats (Maggio et al., 2008).

We hypothesized the substantial role of BBB impairment in the initiation of seizures. Cerebrovascular damage in CNS disorders, including epilepsy is considered as a leading mechanism underlying epileptiform activity (Janigro, 1999; Seiffert, 2004; Marchi et al., 2007; Van Vliet et al., 2015). During intracerebral hemorrhage, blood compounds bleeding into the brain tissue and cause both an acute and a delayed effect on neuronal functioning (Friedman, 2011). Among the consequences of BBB damage are the changes in the intracerebral environment, when the ionic composition of the intercellular cerebrospinal fluid in the affected areas is close in concentration to blood plasma (Zauner et al., 1996; Reinert et al., 2000). Replacing the ACSF with blood plasma saline obviously affects the functioning of voltage-gated channels. Thus, increased concentrations of K^+^ in extracellular solution, in addition to affecting the membrane potential and synaptic transmission, potentiate the persistent Na^+^ currents in neurons (Somjen and Müller, 2000). However, such an effect contributes to the strengthening of synaptic potentials and increases the ability of neurons to recurrent synchronous discharges (Stafstrom, 2007), which we observed in our experiments. Modification of ACSF to blood plasma solution also evolves changes in Ca^2+^ and Mg^2+^ concentrations, which could neutralize the negative surface charges on the outer membrane surface and lead to the facilitation of the voltage-gated channels activation (Isaev et al., 2012). In our study, changes in the concentrations of certain ions in blood plasma media neutralized each other, which contributed to maintaining the shielding of negative charges on neural membranes at the control level. Therefore, we excluded the possibility that the epileptiform activity of the hippocampal slices in modified ACSF is related to changes in the concentrations of divalent cations.

BBB breakdown could result in penetrating and storage in the brain of toxic bloodborne molecules such as hemoglobin, albumin, thrombin, fibrinogen, iron-containing hemosiderin, plasmin, free iron, and environmental toxins (Montagne et al., 2016). Only thrombin was shown to have a potent role in the generation of early-onset SLA (Willmore et al., 1978; Lee et al., 1997; Tomkins et al., 2007). Our data are in agreement with these studies demonstrating the generation of epileptiform activity due to thrombin application in the blood plasma media. Recent findings suggest a significant increase in the thrombin level in the brain tissue caused by the enhancement of BBB permeability during pathological conditions (Woitzik et al., 2011; Isaev et al., 2015). Thrombin, through its major receptor in the neural tissue, protease-activated receptors 1 (PAR 1), produces epileptogenesis by the escalation of brain damage, induction of seizures, inflammation, and neurogenesis (Rohatgi et al., 2004).

It was shown previously that pilocarpine-induced SE may be caused by enhancement in the BBB permeability (Uva et al., 2008). It was shown the disruption of BBB shortly after SE (van Vliet et al., 2007), accompanied by the early efflux of serum proteins and disturbance in interstitial fluid homeostasis (Friedman, 2011). Later epileptic phase involved different mechanisms of propagation seizures, including activation of the innate immune system, activation of transforming growth factor beta in the response to serum albumin, extracellular accumulation of K^+^ and glutamate (Cacheaux et al., 2009; David et al., 2009), etc. In our study, we demonstrate that epileptic activity in slices of pilocarpine-treated rats obtained after SE-onset have similar features as synchronous discharges due to the application of thrombin in plasma ionic media. We propose the essential role of thrombin efflux in the acute epileptiform discharges induced by pilocarpine treatment.

## Data availability statement

The original contributions presented in the study are included in the article, further inquiries can be directed to the corresponding author.

## Ethics statement

The animal study was reviewed and approved by European Convention for the protection of vertebrate animals used for experimental and other scientific purposes (European convention, Strasburg, 1986); the Law of Ukraine “On protection of animals from cruelty” and approved by the Animal Care Committee of Bogomoletz Institute of Physiology.

## Author contributions

AS has designed the experiments, provided an elecrophysiological recordings, made statistical analysis and prepared the manuscript. MK performed experiments with Li-pilocarpine model of epilepsy and took part in disscusion of the results. MS has provided experiments with thrombin and participated in disscusion of manuscript. DI elaborated the idea of experiments, participated in summarizing results and making conclusions. All authors contributed to the article and approved the submitted version.

## Conflict of Interest

The authors declare that the research was conducted in the absence of any commercial or financial relationships that could be construed as a potential conflict of interest.

## Publisher’s note

All claims expressed in this article are solely those of the authors and do not necessarily represent those of their affiliated organizations, or those of the publisher, the editors and the reviewers. Any product that may be evaluated in this article, or claim that may be made by its manufacturer, is not guaranteed or endorsed by the publisher.

## References

[B1] Benveniste H. Drejer J. Schousboe A. Diemer N. H. (1984). Elevation of the extracellular concentrations of glutamate and aspartate in rat hippocampus during transient cerebral ischemia monitored by intracerebral microdialysis. J. Neurochem. 43, 1369–1374. 10.1111/j.1471-4159.1984.tb05396.x 6149259

[B2] Brown R. C. Davis T. P. (2002). Calcium modulation of adherens and tight junction function: a potential mechanism for blood- brain barrier disruption after stroke. Stroke 33, 1706–1711. 10.1161/01.str.0000016405.06729.83 12053015

[B3] Cacheaux L. P. Ivens S. David Y. Lakhter A. J. Bar-Klein G. Shapira M.. (2009). Transcriptome profiling reveals TGF-β signaling involvement in epileptogenesis. J. Neurosci. 29, 8927–8935. 10.1523/JNEUROSCI.0430-09.2009 19605630 PMC2875073

[B4] Cavalheiro E. A. Silva D. F. Turski W. A. Calderazzo-Filho L. S. Bortolotto Z. A. Turski L. (1987). The susceptibility of rats to pilocarpine-induced seizures is age-dependent. Brain Res. 37, 43–58. 10.1016/0165-3806(87)90227-6 3440212

[B5] Chodobski A. Zink B. J. Szmydynger-Chodobska J. (2011). Blood-brain barrier pathophysiology in traumatic brain injury. Transl. Stroke Res. 2, 492–516. 10.1007/s12975-011-0125-x 22299022 PMC3268209

[B6] David Y. Cacheaux L. P. Ivens S. Lapilover E. Heinemann U. Kaufer D.. (2009). Astrocytic dysfunction in epileptogenesis: consequence of altered potassium and glutamate homeostasis? J. Neurosci. 29, 10588–10599. 10.1523/JNEUROSCI.2323-09.2009 19710312 PMC2875068

[B7] Davies D. C. (2002). Blood- brain barrier breakdown in septic encephalopathy and brain tumours. J. Anat. 200, 639–646. 10.1046/j.1469-7580.2002.00065.x 12162731 PMC1570752

[B8] Friedman A. (2011). Blood-brain barrier dysfunction, status epilepticus, seizures and epilepsy: a puzzle of a chicken and egg? Epilepsia 52, 19–20. 10.1111/j.1528-1167.2011.03227.x 21967353 PMC3234990

[B9] Golderman V. Shavit-Stein E. Gera O. Chapman J. Eisenkraft A. Maggio N. (2019). Thrombin and the protease-activated receptor-1 in organophosphate-induced status epilepticus. J. Mol. Neurosci. 67, 227–234. 10.1007/s12031-018-1228-6 30515700

[B10] Greene C. Hanley N. Reschke C. Reddy A. Mäe M. Connolly R.. (2022). Microvascular stabilization via blood-brain barrier regulation prevents seizure activity. Nat. Commun. 13:2003. 10.1038/s41467-022-29657-y 35422069 PMC9010415

[B11] Isaev D. Ivanchick G. Khmyz V. Isaeva E. Savrasova A. Krishtal O.. (2012). Surface charge impact in low-magnesium model of seizure in rat hippocampus. J. Neurophysiol. 107, 417–423. 10.1152/jn.00574.2011 22031777 PMC3349697

[B12] Isaev D. Lushnikova I. Lunko O. Zapukhliak O. Maximyuk O. Romanov A.. (2015). Contribution of protease-activated receptor 1 in status epilepticus-induced epileptogenesis. Neurobiol. Dis. 78, 68–76. 10.1016/j.nbd.2015.03.026 25843668 PMC4682556

[B13] Isaeva E. Hernan A. Isaev D. Holmes G. L. (2012). Thrombin facilitates seizures through activation of persistent sodium current. Ann. Neurol. 72, 192–198. 10.1002/ana.23587 22926852 PMC3430976

[B14] Janigro D. (1999). Blood-brain barrier, ion homeostasis and epilepsy: possible implications towards the understanding of ketogenic diet mechanisms. Epilepsy Res. 37, 223–232. 10.1016/s0920-1211(99)00074-1 10584972

[B15] Katzman R. Pappius H. M. (1973). Brain Electrolytes And Fluid Metabolism. Baltimore: Williams & Wilkins.

[B16] Lee K. R. Drury I. Vitarbo E. Hoff J. T. (1997). Seizures induced by intracerebral injection of thrombin: a model of intracerebral hemorrhage. J. Neurosurg. 87, 73–78. 10.3171/jns.1997.87.1.0073 9202268

[B17] Maggio N. Shavit E. Chapman J. Segal M. (2008). Thrombin induces long-term potentiation of reactivity to afferent stimulation and facilitates epileptic seizures in rat hippocampal slices: toward understanding the functional consequences of cerebrovascular insults. J. Neurosci. 28, 732–736. 10.1523/JNEUROSCI.3665-07.2008 18199772 PMC6670357

[B18] Marchi N. Angelov L. Masaryk T. Fazio V. Granata T. Hernandez N.. (2007). Seizure-promoting effect of blood-brain barrier disruption. Epilepsia 48, 732–742. 10.1111/j.1528-1167.2007.00988.x 17319915 PMC4135474

[B19] Mihály A. Bozóky B. (1984). Immunohistochemical localization of extravasated serum albumin in the hippocampus of human subjects with partial and generalized epilepsies and epileptiform convulsions. Acta Neuropathol. 65, 25–34. 10.1007/BF00689824 6516799

[B20] Montagne A. Toga A. W. Zlokovic B. V. (2016). Blood-brain barrier permeability and gadolinium: benefits and potential pitfalls in research. JAMA Neurol. 73, 13–14. 10.1001/jamaneurol.2015.2960 26524294 PMC4736734

[B21] Obermeier B. Daneman R. Ransohoff R. M. (2013). Development, maintenance and disruption of the blood-brain barrier. Nat. Med. 19, 1584–1596. 10.1038/nm.3407 24309662 PMC4080800

[B22] Oby E. Janigro D. (2006). The blood-brain barrier and epilepsy. Epilepsia 47, 1761–1774. 10.1111/j.1528-1167.2006.00817.x 17116015

[B23] On N. H. Mitchell R. Savant S. D. Bachmeier C. J. Hatch G. M. Miller D. W. (2013). Examination of blood-brain barrier (BBB) integrity in a mouse brain tumor model. J. Neurooncol. 111, 133–143. 10.1007/s11060-012-1006-1 23184143 PMC3555227

[B24] Racine R. J. (1972). Modification of seizure activity by electrical stimulation: II. Motor seizure. Electroencephalogr. Clin. Neurophysiol. 32, 281–294. 10.1016/0013-4694(72)90177-0 4110397

[B25] Rasmussen R. O’Donnell J. Ding F. Nedergaard M. (2020). Interstitial ions: a key regulator of state-dependent neural activity? Prog. Neurobiol. 193:101802. 10.1016/j.pneurobio.2020.101802 32413398 PMC7331944

[B26] Reinert M. Khaldi A. Zauner A. Doppenberg E. Choi S. Bullock R. (2000). High extracellular potassium and its correlates after severe head injury: relationship to high intracranial pressure. Neurosurg. Focus 8:e10. 10.3171/foc.2000.8.1.2027 16924778

[B27] Rohatgi T. Henrich-Noack P. Sedehizade F. Goertler M. Wallesch C. W. Reymann K. G.. (2004). Transient focal ischemia in rat brain differentially regulates mRNA expression of protease-activated receptors 1 to 4. J. Neurosci. Res. 75, 273–279. 10.1002/jnr.10847 14705148

[B28] Seiffert E. (2004). Lasting blood-brain barrier disruption induces epileptic focus in the rat somatosensory cortex. J. Neurosci. 24, 7829–7836. 10.1523/JNEUROSCI.1751-04.2004 15356194 PMC6729929

[B29] Shypshyna M. Savotchenko A. Kuznetsov K. Veselovsky M. (2021). The effect of thrombin in the serum-adapted ionic environment on the induction of epileptiform firing activity of hippocampal cultured neurons. Fiziol. Zh. 67, 3–10. 10.15407/fz67.05.003

[B30] Somjen G. G. Müller M. (2000). Potassium-induced enhancement of persistent inward current in hippocampal neurons in isolation and in tissue slices. Brain Res. 885, 102–110. 10.1016/s0006-8993(00)02948-6 11121535

[B31] Stafstrom C. E. (2007). Persistent sodium current and its role in epilepsy. Epilepsy Curr. 7, 15–22. 10.1111/j.1535-7511.2007.00156.x 17304346 PMC1797888

[B32] Stolp H. B. Dziegielewska K. M. (2009). Role of developmental inflammation and blood-brain barrier dysfunction in neurodevelopmental and neurodegenerative diseases. Neuropathol. Appl. Neurobiol. 35, 132–146. 10.1111/j.1365-2990.2008.01005.x 19077110

[B33] Tomkins O. Friedman O. Ivens S. Reiffurth C. Major S. Dreier J. P.. (2007). Blood-brain barrier disruption results in delayed functional and structural alterations in the rat neocortex. Neurobiol. Dis. 25, 367–377. 10.1016/j.nbd.2006.10.006 17188501

[B34] Tomkins O. Shelef I. Kaizerman I. Eliushin A. Afawi Z. Misk A.. (2008). Blood- brain barrier disruption in post-traumatic epilepsy. J. Neurol. Neurosurg. Psychiatry 79, 774–777. 10.1136/jnnp.2007.126425 17991703

[B35] Uva L. Librizzi L. Marchi N. Noe F. Bongiovanni R. Vezzani A.. (2008). Acute induction of epileptiform discharges by pilocarpine in the *in vitro* isolated guinea-pig brain requires enhancement of blood-brain barrier permeability. Neuroscience 151, 303–312. 10.1016/j.neuroscience.2007.10.037 18082973 PMC2774816

[B36] Van Vliet E. Aronica E. Gorter J. (2015). Blood-brain barrier dysfunction, seizures and epilepsy. Semin. Cell Dev. Biol. 38, 26–34. 10.1016/j.semcdb.2014.10.003 25444846

[B37] van Vliet E. A. da Costa Araújo S. Redeker S. van Schaik R. Aronica E. Gorter J. A. (2007). Blood-brain barrier leakage may lead to progression of temporal lobe epilepsy. Brain 130, 521–534. 10.1093/brain/awl318 17124188

[B38] Vezzani A. Friedman A. (2011). Brain inflammation as a biomarker in epilepsy. Biomark. Med. 5, 607–614. 10.2217/bmm.11.61 22003909 PMC3625731

[B39] Willmore L. J. Sypert G. W. Munson J. B. (1978). Recurrent seizures induced by cortical iron injection: a model of posttraumatic epilepsy. Ann. Neurol. 4, 329–336. 10.1002/ana.410040408 103489

[B40] Woitzik J. Hohenstein A. Hecht N. Juettler E. Schilling L. (2011). Short period of early reperfusion aggravates blood-brain barrier dysfunction during permanent focal ischemia in rats. Transl. Stroke Res. 2, 67–71. 10.1007/s12975-010-0042-4 24323586

[B41] Wu Y. Wu H. Guo X. Pluimer B. Zhao Z. (2020). Blood-brain barrier dysfunction in mild traumatic brain injury: evidence from preclinical murine models. Front. Physiol. 11:1030. 10.3389/fphys.2020.01030 32973558 PMC7472692

[B42] Zauner A. Bullock R. Kuta A. J. Woodward J. Young H. F. (1996). Glutamate release and cerebral blood flow after severe human head injury. Acta Neurochir. Suppl. 67, 40–44. 10.1007/978-3-7091-6894-3_9 8870800

